# Quercetin Potentiates Docosahexaenoic Acid to Suppress Lipopolysaccharide-induced Oxidative/Inflammatory Responses, Alter Lipid Peroxidation Products, and Enhance the Adaptive Stress Pathways in BV-2 Microglial Cells

**DOI:** 10.3390/ijms20040932

**Published:** 2019-02-21

**Authors:** Grace Y. Sun, Runting Li, Bo Yang, Kevin L. Fritsche, David Q. Beversdorf, Dennis B. Lubahn, Xue Geng, James C. Lee, C. Michael Greenlief

**Affiliations:** 1 Biochemistry Department, University of Missouri, Columbia, MO 65211, USA; sung@missouri.edu (G.Y.S.); lirun@health.missouri.edu (R.L.); lubahnd@missouri.edu (D.B.L.); 2 Department of Chemistry, University of Missouri, Columbia, MO 65211, USA; bykm5@mail.missouri.edu; 3 Department of Nutrition and Exercise Physiology, University of Missouri, Columbia, MO 65211, USA; fritschek@missouri.edu; 4 Departments of Radiology, Neurology and Psychological Sciences, and the Thompson Center, University of Missouri, Columbia, MO 65211, USA; beversdorfd@health.missouri.edu; 5 Department of Bioengineering, University of Illinois at Chicago, IL 60607, USA; xgeng6@uic.edu (X.G.); leejam@uic.edu (J.C.L.)

**Keywords:** quercetin, docosahexaenoic acid, 4-hydroxynonenal, microglial cells, lipopolysaccharide, cPLA_2_

## Abstract

High levels of docosahexaenoic acid (DHA) in the phospholipids of mammalian brain have generated increasing interest in the search for its role in regulating brain functions. Recent studies have provided evidence for enhanced protective effects when DHA is administered in combination with phytochemicals, such as quercetin. DHA and quercetin can individually suppress lipopolysaccharide (LPS)–induced oxidative/inflammatory responses and enhance the antioxidative stress pathway involving nuclear factor erythroid-2 related factor 2 (Nrf2). However, studies with BV-2 microglial cells indicated rather high concentrations of DHA (IC_50_ in the range of 60–80 µM) were needed to produce protective effects. To determine whether quercetin combined with DHA can lower the levels of DHA needed to produce protective effects in these cells is the goal for this study. Results showed that low concentrations of quercetin (2.5 µM), in combination with DHA (10 µM), could more effectively enhance the expression of Nrf2 and heme oxygenase 1 (HO-1), and suppress LPS–induced nitric oxide, tumor necrosis factor-α, phospho-cytosolic phospholipase A_2_, reactive oxygen species, and 4-hydroxynonenal, as compared to the same levels of DHA or quercetin alone. These results provide evidence for the beneficial effects of quercetin in combination with DHA, and further suggest their potential as nutraceuticals for improving health.

## 1. Introduction

Docosahexaenoic acid (DHA), in the form of fish oil, is extensively used as a dietary supplement [1]. Studies with animal models demonstrated the effects of dietary DHA to attenuate neurological abnormalities, including neuroinflammation, depressive behavior, and mitigate neuronal damage following cerebral ischemia and other brain injuries [2,3,4,5]. Besides the central nervous system, DHA can also ameliorate disorders in the peripheral system, such as metabolic syndromes, obesity, and cardiovascular diseases [6]. Studies with cultured cells demonstrated the effects of DHA in the suppression of oxidative and inflammatory responses [7,8], as well as the up-regulation of the adaptive cellular stress pathway involving nuclear factor erythroid-2 related factor 2 (Nrf2) [9,10]. However, in most studies with cultured cells, rather high levels of DHA (25–100 µM and IC_50_ in the range of 60-80 µM) were used to achieve these effects. The physiological relevance of using such high levels of DHA in culture conditions has been questioned [11]. In our present study, we only used concentrations of DHA (i.e., 5 and 10 µM) that have been reported in human circulation following oral supplementation with DHA-enriched oil/supplement [12]. Consequently, we sought to determine whether physiologically relevant concentrations of DHA would have similar anti-oxidant and anti-inflammatory effects on microglial cell function, as well as whether these effects can be enhanced by adding other antioxidant-like compounds.

Many botanical polyphenols that are found in fruits and vegetables offer health benefits due to their ability to suppress oxidative and inflammatory responses [13]. Among these, special interest has been placed on quercetin, a natural flavonoid that is abundant in berries, apples, onions, and other foods [14]. Quercetin is used as a dietary supplement and as a nutraceutical for improving memory and cognition, and for its anti-allergic immune responses [14,15,16,17,18,19]. Studies with cell models also indicated the ability for quercetin to enhance the Nrf2 pathway and increase the production of heme oxygenase-1 (HO-1), which is a potent antioxidative enzyme [20,21,22,23].

Our previous studies demonstrated ability for primary microglia, isolated from mouse brain, to respond to lipopolysaccharide (LPS) stimulation of inflammatory pathways, especially those involving nuclear factor kappa B (NF-κB) and NADPH oxidase [24]. LPS can also stimulate phospho-ERK1/2 (p-ERK1/2) and phospho-cytosolic phospholipase A_2_ (p-cPLA_2_) pathways in primary and BV-2 microglial cells [24]. The potent electrophilic properties of quercetin provide strong evidence for this polyphenol as a candidate for testing the combination effects with DHA. A major goal for this study is to determine whether quercetin, together with DHA can offer additional protective effects, and thus promote this combination as a better supplement or nutraceutical for advancing human health.

## 2. Results

**Figure 1:**
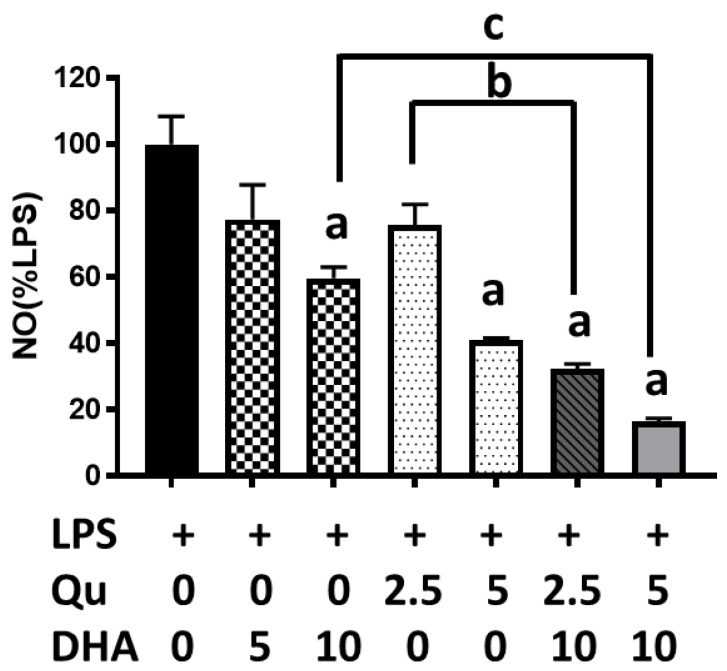
Effects of docosahexaenoic acid (DHA) (5 and 10 μM) and/or quercetin (2.5 and 5 μM) on lipopolysaccharide (LPS)-induced nitric oxide (NO) production in microglial cells. NO was measured using the Griess reaction protocol. NO data obtained from cells without LPS were subtracted from cells with LPS, and the differences were regarded as 100%. Results from three individual cell passages with triplicate assays from each passage are expressed as the mean ± SEM (*n* = 3) and analyzed by one-way ANOVA followed by Tukey’s post-tests. “a” represents significant differences (*p* < 0.01) between control versus LPS. “b” and “c” represent significant differences (*p* < 0.05) between the indicated pairs.

In the initial experiment, we tested the effects of DHA at 5 and 10 µM and quercetin at 2.5 and 5 µM on the inhibition of LPS-induced nitric oxide (NO) production in microglial cells. As shown in Figure 1, results showed significant (*p* < 0.05) inhibition by DHA at 10 µM and quercetin at 5 µM. However, quercetin at 2.5 and 5 µM together with DHA at 10 µM showed significantly (*p* < 0.0001) stronger inhibition as compared with treating cells with the same levels of DHA or quercetin alone. For subsequent studies, a combination of quercetin at 2.5 µM and DHA at 10 µM was used to test the effects on oxidative and inflammatory effects on BV-2 microglial cells.

**Figure 2:**
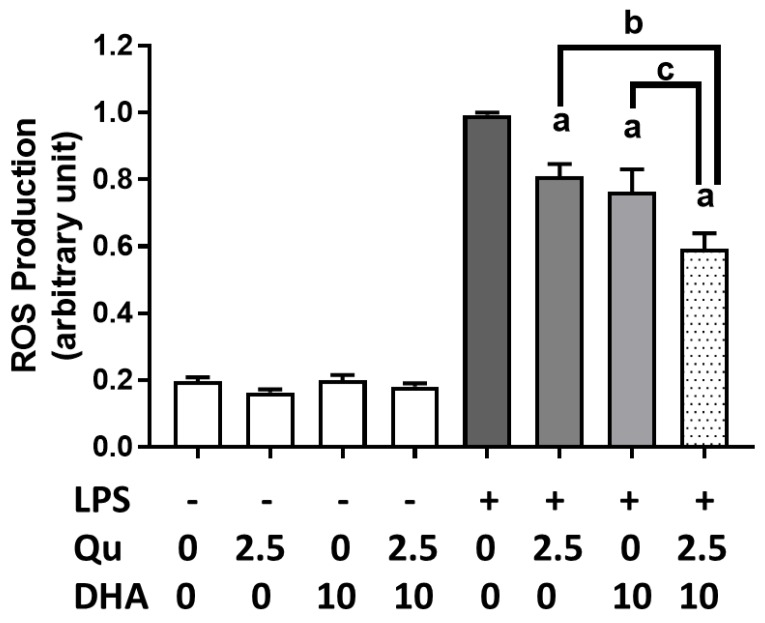
Effects of DHA and/or quercetin on LPS-induced reactive oxygen species (ROS) production in microglial cells. ROS was detected using CM-H_2_DCFDA (DCF), as described in text. “a” represents significant differences (*p* < 0.01) between control versus treatments. “b” and “c” represent significant differences (*p* < 0.05) between the indicated pairs.

Using the CM-H_2_DCFDA, we examined the effects of DHA (10 µM) and/or quercetin (2.5 µM) on reactive oxygen species (ROS) production in microglial cells incubated with or without LPS (100 ng/mL). As shown in Figure 2, DHA or quercetin alone significantly suppressed LPS-induced ROS production, but the combination of quercetin and DHA produced significantly (*p* < 0.0001) stronger inhibitory effects. Treatment with DHA and/or quercetin without LPS did not alter endogenous ROS levels in the cells.

**Figure 3:**
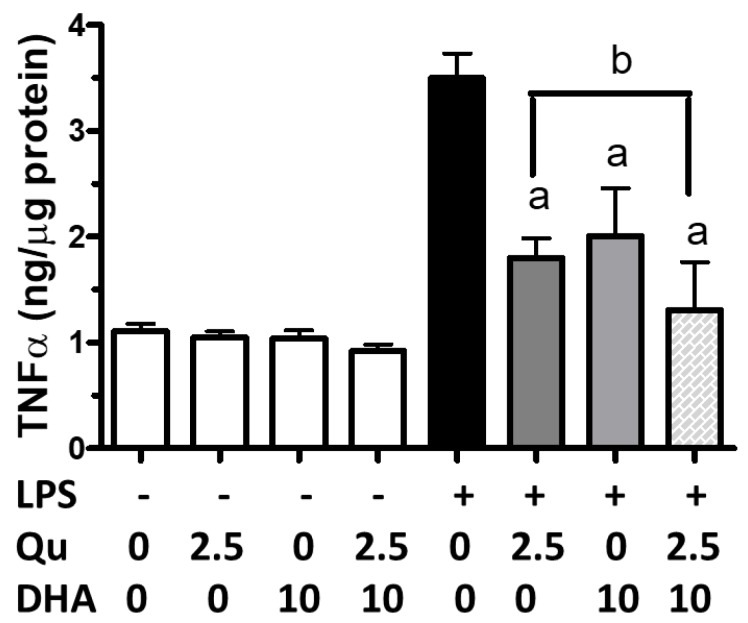
Effects of DHA and/or quercetin on LPS-induced tumor necrosis factor-α (TNFα) in microglial cells. TNFα using the ELISA kit was described in text. “a” represents significant differences (*p* < 0.01) between control versus treatments. “b” represents significant differences (*p* < 0.05) between the indicated pair.

An ELISA protocol was used to determine the effects of quercetin and/or DHA on LPS-induced tumor necrosis factor-α (TNFα). As shown in Figure 3, although DHA (10 µM) and quercetin (2.5 µM) could significantly (*p* < 0.05) inhibit LPS-induced TNFα, a combination of DHA and quercetin showed significantly (*p* < 0.001) greater reduction when compared with DHA and quercetin alone.

**Figure 4:**
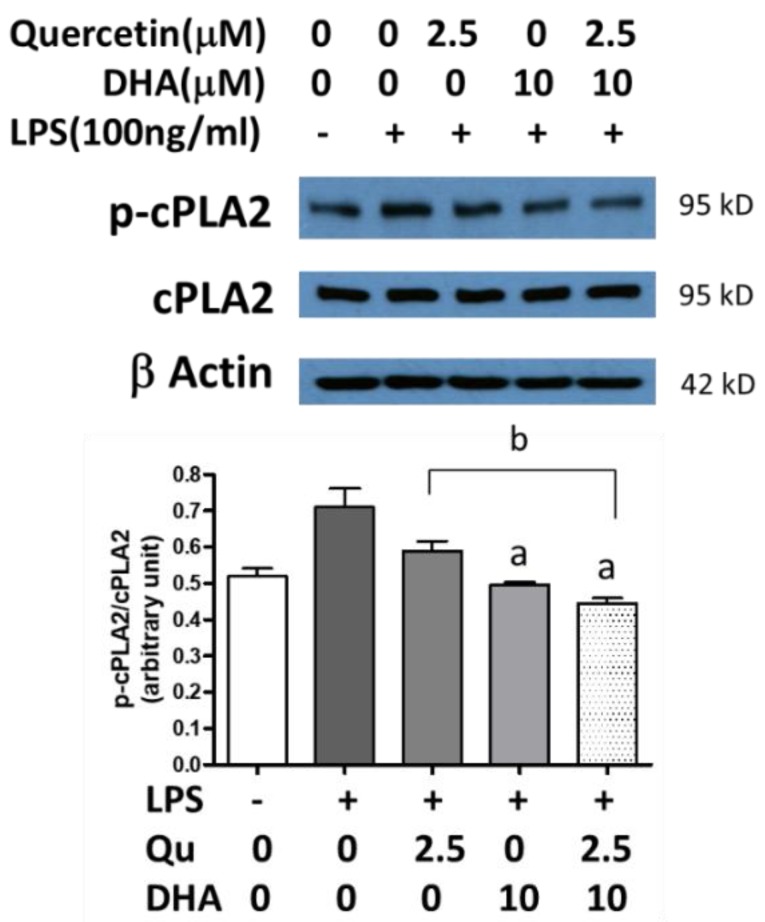
Effects of DHA and/or quercetin on LPS-induced phospho-cytosolic phospholipase A_2_ (p-cPLA_2_) expression in microglial cells. Data represent p-cPLA_2_/cPLA_2_ ratios from three individual experiments. “a” represents significant differences (*p* < 0.01) between control versus treatments. “b” represents significant differences (*p* < 0.05) between the indicated pairs.

In Figure 4, the p-cPLA_2_/cPLA_2_ ratio was measured in microglial cells under a variety of conditions. The endogenous levels gave a ratio of about 0.5. The addition of LPS resulted in a higher p-cPLA_2_/cPLA_2_ ratio. Adding 2.5 µM quercetin to the LPS-stimulated cells decreased the ratio, as did the addition of 10 µM DHA. The combination of DHA and quercetin decreased the p-cPLA_2_/cPLA_2_ ratio to below endogenous levels (*p* < 0.01).

**Figure 5:**
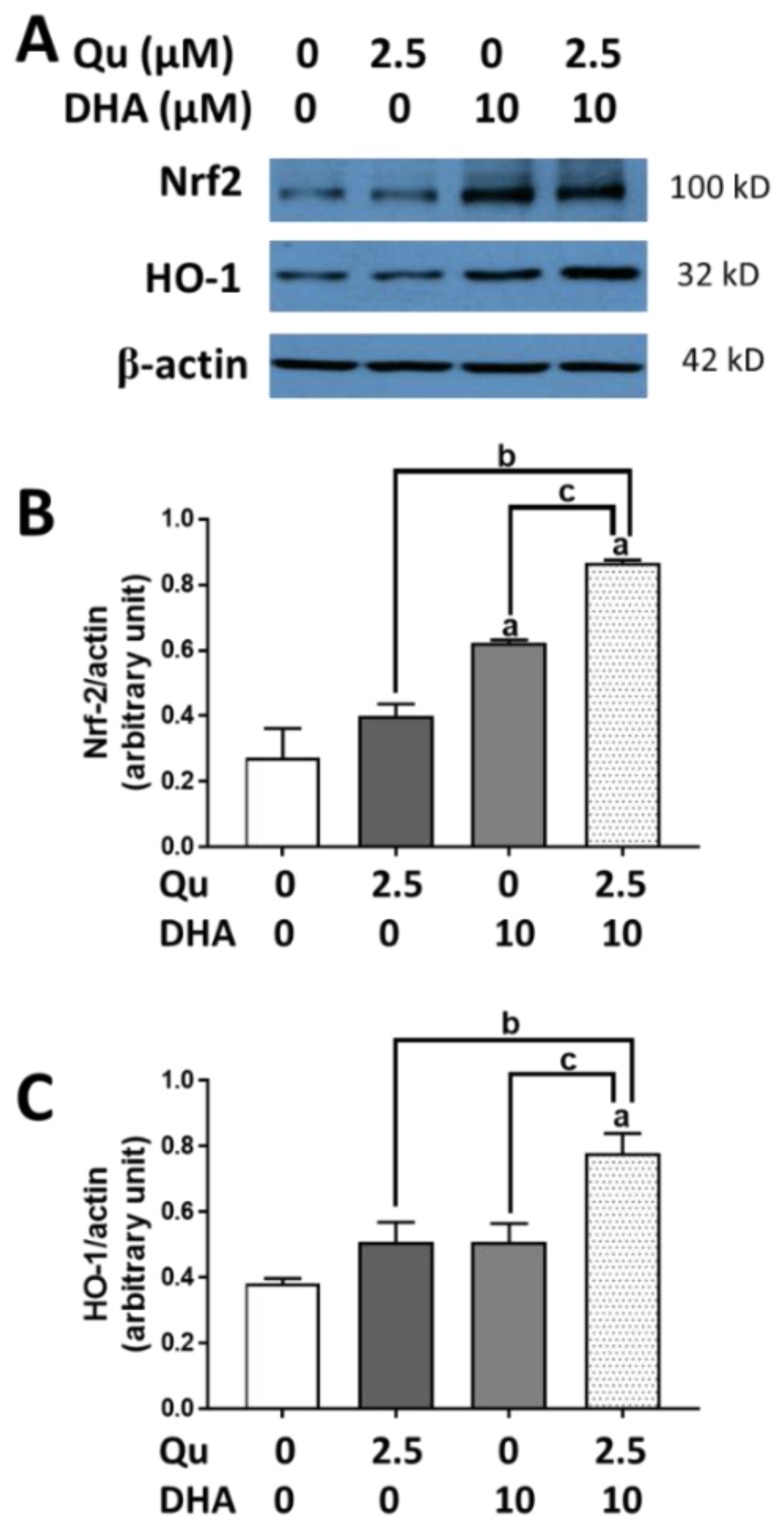
Effects of DHA and/or quercetin on induction of Nrf2 and HO-1 protein expression in BV-2 microglial cells. (**A**) A representative Western blot showing effects of DHA and/or quercetin on Nrf2 and HO-1 expression with β-actin as loading control. (**B**,**C**) Bar graphs represent relative densities of Nrf2/β-actin and HO-1/β-actin ratios from three experiments with different passages. “a” represents significant differences (*p* < 0.01) comparing between control versus treatment. “b” and “c” represent significant differences (*p* < 0.05) between the indicated pairs.

Besides suppressing oxidative and inflammatory responses, quercetin and DHA have been shown to enhance the Nrf2 pathway and the induction of HO-1 in microglial cells [20]. In the present study, although DHA (10 µM) or quercetin (2.5 µM) could individually increase the expression of Nrf2 and HO-1, a significantly greater effect (*p* < 0.01) was observed when quercetin was added together with DHA (Figure 5A–C).

**Figure 6:**
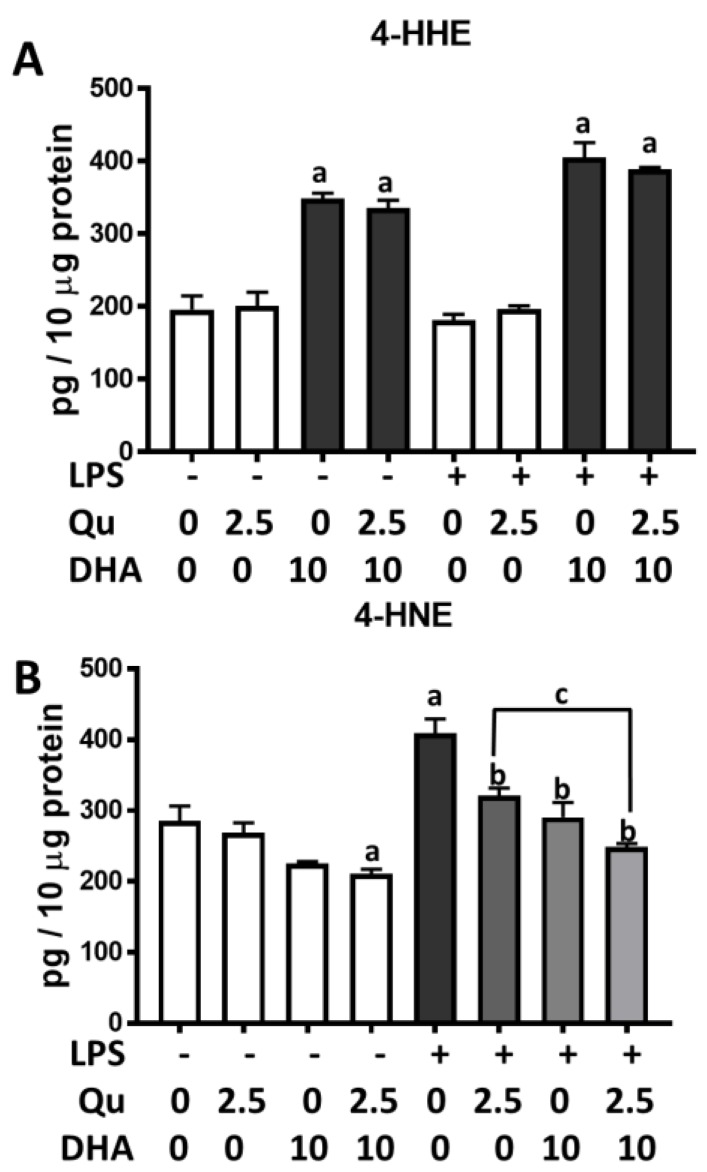
Effects of DHA and/or quercetin on 4-hydroxyhexenal (4-HHE) and 4-hydroxynonenal (4-HNE) levels in microglia cells. Levels of 4-HHE (**A**) and 4-HNE (**B**) upon treatment with DHA and/or quercetin in the presence and absence of LPS (100 ng/mL). “a” represents significant differences (*p* < 0.0001) comparing between control versus each treatment. “b” represents significant differences (*p* < 0.01) comparing between LPS versus each treatment. “c” represents significant differences (*p* < 0.05) between the indicated pairs.

In this study, we tested the effects of quercetin and DHA on lipid peroxidation products. As shown in Figure 6A, cells that were treated with DHA (10 µM) for 7 h showed a significant increase in levels of 4-hydroxyhexenal (4-HHE). Quercetin did not alter the increase in 4-HHE induced by DHA. The stimulation of cells with LPS (100 ng/mL) resulted in a significant (*p* < 0.05) increase in 4-hydroxynonenal (4-HNE) levels as compared with the control (Figure 6B). Under this condition, both quercetin (2.5 µM) or DHA (10 µM) significantly (*p* < 0.001) suppressed LPS-induced 4-HNE production, and a combination of quercetin and DHA more effectively suppressed 4-HNE production as compared with quercetin and DHA alone (Figure 6B).

## 3. Discussion

**Figure 7:**
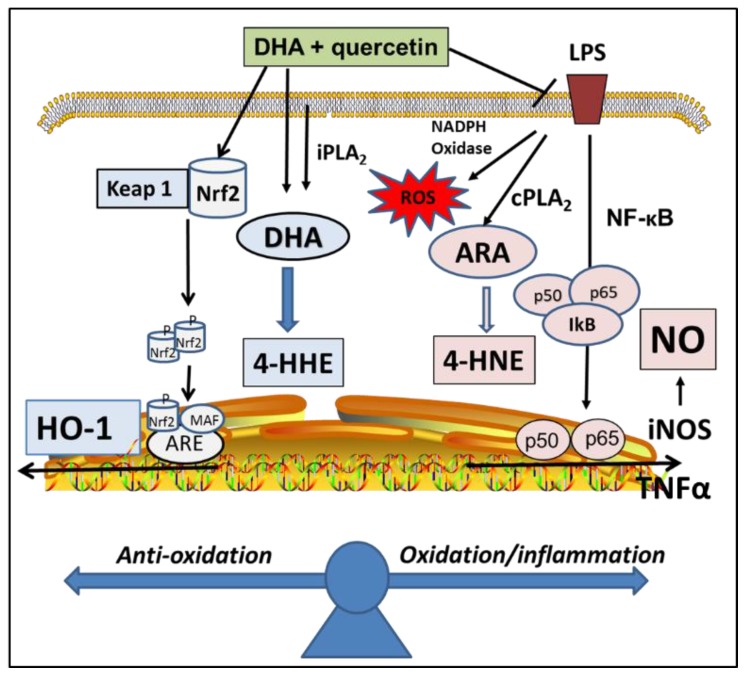
An integrated scheme depicting effects of DHA and quercetin on up-regulation of the Nrf2/HO-1 pathway and down-regulation of NF-κB/TNFα/cPLA_2_/ROS pathways, and metabolic pathways for production of 4-HHE and 4-HNE through lipid peroxidation mechanisms.

This study demonstrated that treating BV-2 microglial cells with quercetin (2.5 µM), together with DHA (10 µM), could provide stronger protective effects as compared with treating with the same concentrations of quercetin or DHA alone. These levels of quercetin and DHA were much lower when compared to the levels used in our previous studies [7,20]. The protective effects in the present study included an attenuation of LPS-induced inflammatory and oxidative pathways, and an enhancement of the Nrf2/HO-1 pathway. Here, LPS induced NO and TNFα were used to represent the activation of the NF-κB transcriptional pathway [25]. The stimulation of cPLA_2_ is regarded an important inflammatory pathway, because the release of arachidonic acid (ARA) contributes to synthesis of eicosanoids, which are inflammatory mediators [24]. Besides the suppression of NO, ROS, TNFα, and cPLA_2_ pathways, quercetin in combination of DHA also showed a significantly greater enhancement in the expression of Nrf2 and HO-1, which are examples of the adaptive cellular anti-oxidative/stress pathway (Figure 7). Based on the potent electrophilic property of quercetin, we believe that a unique advantage for this combination is due partly to the ability for quercetin to enhance the Nrf2 pathway [20]. Our results are in agreement with a study by Si et al., who, in 2016, demonstrated the ability for quercetin to enhance DHA’s ability to suppress LPS-induced NF-κB inflammatory effects in macrophages [26]. However, our results here included effects of the Nrf2/HO-1 pathway as well as levels of lipid peroxidation products. With these results, it is reasonable to design future studies to test whether such a combination may produce additive effects with animal studies.

There is increasing evidence for studies whereby nutraceuticals are formulated by combining phytochemicals with n-3 polyunsaturated fatty acids (PUFAs). For example, the administration of Q-Mix (a nutraceutical comprising quercetin and fish oil) to adult obese women in a random trial showed beneficial effects against inflammation as well as enhanced immune functions [27]. In another study with macrophages, DHA combined with low dose of curcumin, a polyphenol from turmeric, was shown to offer additional protective effects against inflammatory and oxidative responses [28]. Similar to our study with quercetin, the effects of curcumin were attributed to its ability to enhance the Nrf2 antioxidant stress pathway. Another study showed that dietary fish oil, together with apple polyphenol, could decrease serum cholesterol levels in rats [29]. Besides studies on the peripheral system, there is evidence that n-3 PUFAs, together with uridine and choline, could improve age-related decline in synaptic function and cognitive function, which carries implications for Alzheimer’s disease [30,31]. Taken together, these studies support additional beneficial effects when n-3 PUFAs are administered in combination with phytochemicals with anti-oxidant and electrophilic properties and, among these, quercetin is an excellent candidate for combination with DHA.

Our recent studies with the BV-2 microglial cells demonstrated that, while the supplementation of cells with DHA led to an increase in 4-HHE, an increase in 4-HNE levels could be produced through LPS stimulation of the cPLA_2_/ARA pathway [7]. Based on these findings, it is reasonable to link 4-HNE to the inflammatory responses and 4-HHE to the anti-inflammatory responses (Figure 7). In this study, quercetin, together with DHA, was shown to more effectively suppress LPS-induced 4-HNE as compared with treating with DHA and quercetin alone. On the other hand, treating cells with quercetin did not alter DHA-induced increased in 4-HHE levels [7]. Studies with other cells and animal models also provided evidence for electrophilic properties of 4-HHE and 4-HNE, and when applied exogenously to cells, both can enhance the Nrf2/HO-1 pathway [3,7,32]. Whether the increase in 4-HHE and the decrease in LPS-induced 4-HNE due to treating cells with quercetin and DHA results in an alteration of cell redox equilibrium remains an important question that requires further study.

## 4. Materials and Methods

### 4.1. Materials

Dulbecco’s modified Eagle’s medium (DMEM) and penicillin/streptomycin were obtained from GIBCO (Gaithersburg, MD, USA). 4-hydroxyhexenal (4-HHE), 4-hydroxynonenal (4-HNE), 4-hydroxyhexenal-d_3_ (4-HHE-d_3_), and docosahexaenoic acid (DHA) were purchased from Cayman Chemical (Ann Arbor, MI, USA). Quercetin (> 95%), fetal bovine albumin (FBS), Greiss reagent (sulfanilamide and N-1-napthylethylenediamine dihydrochloride), 1,3-cyclohexanedione (CHD, 97%), ammonium acetate (HPLC grade), acetic acid (ACS grade), and formic acid (mass spectrometry grade) were purchased from Sigma-Aldrich (St. Louis, MO, USA). CM-H_2_DCFDA was purchased from Invitrogen (Eugene, OR, USA). The antibodies used include the following: Nrf2 and HO-1 from Santa Cruz Biotechnology (Santa Cruz, CA, USA), p-cPLA_2_ and cPLA_2_ from Cell Signaling (Beverly, MA, USA), and monoclonal anti-β-actin peroxidase from Sigma-Aldrich (St. Louis, MO, USA). Mouse uncoated ELISA kit for TNFα was purchased from Invitrogen (St. Louis, MO, USA). C18 Sep-Pak cartridges were obtained from Waters Corporation (Milford, MA, USA). Phospholipid removal cartridges (Phree^TM^, 1 mL) were purchased from Phenomenex (Torrance, CA, USA). All of the solvents (HPLC grade) that were used for LC and MS analysis were obtained from Thermo Fisher Scientific (Fair Lawn, NJ, USA).

### 4.2. Cell Culture and Treatments

BV-2 microglial cells were originally cultured in 75 cm^2^ flasks with DMEM that was supplemented with 5% FBS containing 100 units/mL penicillin and streptomycin (100 µg/mL) in a 5% CO2 incubator at 37 °C. The cells were normally used between 14–25 passages [33]. For experiments, cells were subcultured in 96- or 12-well plates to 80–90% confluence and then serum starved for 3 h prior to adding quercetin and/or DHA for 1 h, and followed by stimulation with LPS (100 ng/mL) for the indicated time. DHA and quercetin were initially prepared in DMSO and subsequently diluted with DMEM to the required concentrations during experiment. The controls contained a similar amount of DMSO and this amount did not alter cell viability using the WST-1 assay.

### 4.3. NO Determination in Culture Medium

NO released from cells was converted to nitrite in the culture medium and it was determined using the Griess reagent [25]. Briefly, cells in 96 well-plates were serum starved, treated with DHA and/or quercetin for 1 h, and then stimulated with LPS for 16 h. Aliquots (50 µL) of culture medium were incubated with 50 µL of reagent A (1%, *w*/*v*, sulfanilamide in 5% phosphoric acid) for 10 min at room temperature in the dark, followed by incubation with 50 µL of reagent B (0.1%, *w*/*v*, N-1-napthylethylenediamine24 h dihydrochloride) for 10 min at room temperature in the dark. The absorbance of samples was measured at 565 nm using the Synergy4 Plate Reader (BioTek Instruments, St. Louis, MO, USA). Sodium nitrite (0–100 µM), diluted in culture media, was used to prepare the nitrite standard curve.

### 4.4. Measurement of ROS Production

ROS production was measured using the ROS detection reagent CM-H_2_DCFDA (DCF), as described previously [34]. Briefly, cells in 96 well-plates were serum starved for 3 h in prior to adding quercetin and DHA for 1 h and followed by the addition of LPS for 11 h. DCF (1 µM final concentration) was added to each well for 1 h. The fluorescence intensity of DCF was measured using the Synergy4 Plate Reader with an excitation wavelength of 490 nm and an emission wavelength of 520 nm.

### 4.5. Determination of TNFα

The experimental design for measurement of TNFα was similar to that for the ROS determination. Cells were cultured in 24-well plates and after being serum starved for 3 h, quercetin (2.5 µM) and/or DHA (10 µM) were added for 1 h, followed by stimulation with or without LPS (100 ng/mL) for 16 h. After treatment, an aliquot of the supernatant was transferred to Eppendorf tube and then centrifuged at 4000× *g* for 5 min. The remaining cells were lysed with RIPA buffer and saved for protein determination with BCA. ELISA for measurements of TNFα was conducted according to manufacturer’s protocol. Samples were read at 450 nm and subtracted the values at 570 nm.

### 4.6. Western Blot Analysis

Western blot analysis was carried out as described earlier [24]. Briefly, the cells were cultured in 12 well plates and after treatment, Laemmli lysis buffer was added, and the lysate was centrifuged at 10,000× *g* for 15 min at 4 °C to remove cell debris. An aliquot was used for protein quantification with the BCA protein assay kit. Samples, together with molecular weight standard, were loaded on sodium dodecyl sulfate-polyacrylamide gel and electrophoresis was carried out at 100 V. After electrophoresis, the proteins in the gel were transferred to 0.45 µm nitrocellulose membranes at 100 V for 1.5 h. The membranes were blocked in Tris-buffered saline, pH 7.4, with 0.1% Tween 20 (TBS-T) containing 5% non-fat milk for 1.5 h at room temperature. The following antibodies were applied: anti-Nrf2 (1:500 dilution), anti-HO-1 (1:800 dilution), anti-p-cPLA_2_ (1:1000 dilution), and anti-cPLA_2_ (1:1000 dilution) overnight at 4 °C. After repeated washing with TBS-T, the blots were incubated with goat anti-rabbit IgG-horseradish peroxidase (1:6000 dilution) for 1 h at room temperature. After washing with TBS-T, immuno-labeling was detected by exposing to SuperSignal chemiluminescent substrates (Thermo Scientific, Rockford, IL, USA). For loading control, the blots were incubated with anti-β-actin (1:50,000) and goat anti-mouse IgG-horseradish peroxidase (1:6000). Films were scanned and the optical density of the protein bands was measured using the QuantityOne software program (BioRad, Hercules, CA, USA).

### 4.7. LC-MS/MS Analysis of 4-HHE and 4-HNE in Microglial Cells

LC-MS/MS analysis was carried out as described earlier [7]. Briefly, cells were subcultured in 60 mm dishes, and after different treatment conditions, the medium was removed and 0.5 mL of phosphate-buffered saline-methanol (1:1, *v*/*v*) was added. An aliquot of cell suspension was added to equal volume of internal standard (4-HHE-d_3_, 1000 ng/mL) and acetonitrile (0.5 mL) containing 1% formic acid was added to the mixture. Solid phase extraction (SPE) was carried out using a Phree^TM^ cartridge. 4-HHE, 4-HNE, and 4-HHE-d_3_ were derivatized by adding 200 µL of acidified 1,3-cyclohexanedione reagent at 60 °C for 1 h. The derivatized 4-HHE and 4-HNE were desalted using a C18 SPE cartridge and the eluate evaporated to dry. An aliquot of the reconstituted solution was injected into a Waters Xevo TQ-S triple quadrupole mass spectrometer. The multiple reaction monitoring transitions *m*/*z* 326.3 > 216.1 Da, 284.2 > 216.1 Da, and 287.2 > 216.1 Da were chosen for the simultaneous monitoring of the 4-HNE, 4-HHE, and 4-HHE-d_3_ derivatives, respectively. MassLynx software (v4.1, Waters) was used for all data acquisition.

### 4.8. Statistical Analysis

For studies to assess oxidative and inflammatory responses, triplicate analyses were performed on a given sample and at least three independent experiments with different passages were performed for each condition. For studies measuring 4-HHE and 4-HNE levels using the LC-MS/MS analysis, experiments were carried out with three biological replicates and two analytical replicates. The results are expressed as the mean ± standard error of the mean (SEM) and analyzed by one-way ANOVA, followed by Tukey’s post-tests (v8.01; GraphPad Prism Software, San Diego, CA, USA). The differences were considered to be significant at *p* < 0.05 for all analyses.

## 5. Conclusions

Results from this study provided evidence to support additive protective effects upon the addition of quercetin in combination to DHA in microglial cells. The combined effects are consistent with them being at least additive. Future studies can use this combination to test animal models and determine whether the mixture can be used to develop novel nutraceuticals for improving human health.

## Abbreviations

ANOVA	Analysis of variance
ARA	Arachidonic acid
BEL	Bromoenol lactone
CHD	1,3-cyclohexanedione
CM-H_2_DCFDA	5-(and-6)-chloromethyl-2′,7′-dichlorodihydrofluorescein diacetate, acetyl ester
cPLA_2_	Cytosolic phospholipase A_2_
DHA	Docosahexaenoic acid
DMEM	Dulbecco’s modified Eagle’s medium
DMSO	Dimethyl sulfoxide
ERK	Extracellular signal-regulated kinases
ESI	Electrospray ionization
FBS	Fetal bovine serum
4-HHE	4-Hydroxy hexenal
4-HHE-d_3_	4-Hydroxy hexenal-d_3_
4-HNE	4-Hydroxy nonenal
HO-1	Heme oxygenase 1
iPLA_2_	Calcium-dependent phospholipase A_2_
LC-MS/MS	Liquid chromatography tandem mass spectrometry
LPS	Lipopolysaccharide
MRM	Mutliple reaction monitoring
NF-κB	Nuclear factor kappa-light-chain-enhancer of activated B cells
NO	Nitric oxide
Nrf2	Nuclear factor erythroid-2 related factor 2
PLA_2_	Phospholipase A_2_
PUFAs	Polyunsaturated fatty acids
ROS	Reactive oxygen species
SEM	Standard error of the mean
SPE	Solid phase extraction
TBS	Tris-buffered solution
TNF-α	Tumor necrosis factor α
